# Organization and Regulation of Soybean SUMOylation System under Abiotic Stress Conditions

**DOI:** 10.3389/fpls.2017.01458

**Published:** 2017-08-21

**Authors:** Yanjun Li, Guixin Wang, Zeqian Xu, Jing Li, Mengwei Sun, Jingsong Guo, Wei Ji

**Affiliations:** Department of Biotechnology, College of Life Science, Northeast Agricultural University Harbin, China

**Keywords:** soybean, SUMO pathway, expression profiling, abiotic stress, SUMOylation

## Abstract

Covalent attachment of the small ubiquitin-related modifier, SUMO, to substrate proteins plays a significant role in plants under stress conditions, which can alter target proteins' function, location, and protein-protein interactions. Despite this importance, information about SUMOylation in the major legume crop, soybean, remains obscure. In this study, we performed a bioinformatics analysis of the entire soybean genome and identified 40 genes belonged to six families involved in a cascade of enzymatic reactions in soybean SUMOylation system. The *cis*-acting elements analysis revealed that promoters of SUMO pathway genes contained different combinations of stress and development-related *cis*-regulatory elements. RNA-seq data analysis showed that SUMO pathway components exhibited versatile tissue-specific expression patterns, indicating coordinated functioning during plant growth and development. qRT-PCR analysis of 13 SUMO pathway members indicated that majority of the SUMO pathway members were transcriptionally up-regulated by NaCl, heat and ABA stimuli during the 24 h period of treatment. Furthermore, SUMOylation dynamics in soybean roots under abiotic stress treatment were analyzed by western blot, which were characterized by regulation of SUMOylated proteins. Collectively, this study defined the organization of the soybean SUMOylation system and implied an essential function for SUMOylation in soybean abiotic stress responses.

## Introduction

The conjugation of protein tags or modifiers to target proteins is one important protein post-translational modification for the maintenance of cellular homeostasis and biological activities in eukaryotes during rapid environmental changes, when physiological responses often occur both quickly and reversibly. In recent years, the small ubiquitin-related modifier (SUMO) proteins of approximately 100–115 amino acids, has emerged as an influential class of protein modifiers which has been shown to regulate various nuclear processes, including transcriptional control, subcellular trafficking, and regulation of the cell cycle (Miller et al., 2010, 2013; Park H. C. et al., 2011).

The modification process of SUMOylation is mechanistically similar to the conjugation of ubiquitin, the most prominent representative of small protein modifiers with conserved structure (Rodriguez et al., 2001; Matic et al., 2010). SUMO is synthesized as a pre-protein that needs to be processed by ubiquitin-like proteases (ULP) to expose a carboxyl-terminal double glycine (di-Gly) motif. Analogous to ubiquitination, SUMO conjugation in plants is facilitated through a multi-step enzymatic pathway including E1 enzymes for SUMO activation, E2 enzymes for conjugation and E3 enzymes for ligation (Castro et al., 2012). The end result is SUMO isopeptide conjugated via its C-terminal diGly motif to accessible lysine (Lys) within substrate proteins. Interestingly, SUMOylation is a reversible process, and de-SUMOylation is accomplished through ULP class of SUMO proteases which release free SUMOs from protein conjugates for further conjugation cycles (Colby et al., 2006; Mukhopadhyay and Dasso, 2007).

Unlike ubiquitylation, SUMOylation is not typically committing proteins to breakdown by the 26S proteasome, but can change target protein localization, repress or enhance activity of target transcription factor, regulate interactions between proteins, and mediate cross talk with other post-translational modifications (Bossis and Melchior, 2006). SUMO conjugation was reported to be essential in *Arabidopsis*, and an increasing number of papers have demonstrated that mutants that were not able to attach SUMO1/2 onto substrate proteins exhibit typical phenotypes such as growth reduction, impaired salinity, drought, heat and freezing tolerance, and altered response to phosphate starvation (Roden et al., 2004; Catala et al., 2007; Miura et al., 2007b, 2011; van den Burg et al., 2010). Previous studies with rice (*Oryza sativa*) and poplar (*Populus strichocarpa*) showed that SUMOylation can be significantly enhanced upon heat, cold, high salinity, and abscisic acid exposure (Chaikam and Karlson, 2010; Reed et al., 2010; Li et al., 2013), which was always accompanied by a decrease in the pool of free SUMO and correlated with the duration and intensity of the stress. Interestingly, SUMOylation levels decrease within hours or even minutes when the heat shocked plants returned to the normal temperature, suggesting that SUMOylation acts transiently and reversibly (Kurepa et al., 2003; van den Burg et al., 2010). Recently, SUMOylation was reported to display a striking memory that dampened succeeding conjugate increases if a second stimulus was provided too close to the first one (Kurepa et al., 2003; Miller et al., 2013).

Further genetic studies provided evidence that several members of SUMOylation system contributed to the regulation of development and adaptation to abiotic stress conditions. SUMO1 and SUMO2 in *Arabidopsis* appeared to be functionally redundant, but elimination of them was embryo lethal (Park H. J. et al., 2011). On the other hand, over-expression of either *AtSUMO1* or *AtSUMO2* gene was correlated with ABA-mediated growth inhibition (Tatham et al., 2001; Aguilar-Martinez et al., 2015). Besides, null E3 ligase *SIZ1* alleles (*siz1-1, siz1-2*, and *siz1-3*) in *Arabidopsis* displayed several phenotypes including hypersensitive to extreme temperature and drought stress, as well as enhanced tolerance to salinity stress, indicating that *SIZ1*-mediated SUMOylation play an important role in response to abiotic stress (Miura et al., 2007b, 2011; Cheong et al., 2009; Chen et al., 2011). Moreover, *Arabidopsis* mutants lacking SUMO proteases *OTS1* and *OTS2* showed inhibition of root growth under salt treatment (Conti et al., 2008), while transgenic rice plants over-expressing *OsOTS1* gene exhibited increased salt tolerance concomitant with reduced levels of SUMOylated proteins (Srivastava et al., 2016). It indicated that manipulation of conjugation or de-conjugation of SUMO from target proteins could be an effective method in plants to cope with stress conditions.

Soybean is one of the most important legume crops in the world, offering high-quality protein and vegetable oil for human and animal consumption, but the growth and development of soybean seedlings is always affected by adverse conditions (Nakashima et al., 2009; Schmutz et al., 2010). Given the potential importance of SUMOylation to stress defense, presented here is a comprehensive description of core components of SUMO system in soybean. We also investigated the responses of SUMOylation system under salt, high temperature and abscisic acid stress conditions both at transcriptional and translational levels. Our results may suggest a role for SUMOylation in soybean abiotic stress responses, and provide valuable information for further functional studies of SUMOylation cascade components in soybean.

## Materials and methods

### Identification and analysis of soybean SUMO pathway genes

Soybean SUMO pathway genes were identified by tblastn searches against the soybean genome at Phytozome v11.0 (https://phytozome.jgi.doe.gov/pz/portal.html), using the amino acid sequences of the known pathway genes from *Arabidopsis* as queries. The molecular weight and isoelectric point (pI) of each gene were calculated by ExPASy (http://www.expasy.org/tools/). Protein subcellular localization was predicted by the WoLF PSORT (http://www.genscript.com/wolf-psort.html) (Horton et al., 2007).

Multiple sequence alignments of SUMO pathway components in soybean, *Arabidopsis*, poplar, rice, maize and tomato were carried out using clustalW with default parameters (Thompson et al., 2002). The phylogenetic tree was constructed by MEGA (V7.0.20) using the neighbor-joining (NJ) method with the following parameters: Poisson correction, pair-wise deletion and 1000 bootstrap replicates (Tamura et al., 2007). Protein domains were predicted using Pfam (http://pfam.xfam.org) and visualized using BoxShade online software (http://www.ch.embnet.org/software/BOX_form.html) (Beitz, 2000). In addition, possible SUMOylation sites and SIM sequences were predicted using the medium settings of program GPS-SUMO v1.0.1 (Zhao et al., 2014).

### *Cis*-acting elements analysis in promoters of the soybean SUMO pathway genes

PlantCARE online program (http://bioinformatics.psb.ugent.be/webtools/plantcare/html; Lescot et al., 2002) was used to predict *cis*-acting elements in promoters of soybean SUMO pathway genes. Sequence of 1,500 bp upstream of the start codon downloaded from Phytozome 11.0 database was used for *cis*-acting elements scan.

### RNA-Seq atlas analysis of SUMO pathway genes in different tissues

The normalized transcriptome data (Reads/Kb/Millon, RPKM) of 14 soybean samples from six different tissues harvested at different growth periods was downloaded from the SoyBase website (https://soybase.org/soyseq/). These samples include young leaf, flower, one cm pod, pod-shell 10-DAF (days after flowering), pod-shell 14-DAF, root, nodule and developing seeds (seed harvested at 10-DAF, 14-DAF, 21-DAF, 25-DAF, 28-DAF, 35-DAF, and 42-DAF) (Severin et al., 2010). The criteria of expressed gene was the RPKM value equal to or greater than two in the expression atlas (Belamkar et al., 2014). The RPKM normalized read count data of expressed gene was then log_2_-transformed and displayed in the form of heatmap using the OmicShare tools (http://www.omicshare.com/tools).

### Plant growth and treatments

Soybean seeds (*Glycine max* cv Dongnong 50) were germinated on two layers of filter paper soaked in distilled water at 22°C in the dark. After 2 days, germinated seedlings were transferred to vermiculite and irrigated with half-strong modified Hoagland nutrient solution (pH 5.8) in a growth chamber under normal conditions (25/21°C day/night temperature, relative humidity of 60–80% and 16 h light period/day at intensity of 120 μmol photons m^−2^ s^−1^) for 2 weeks. When the second trifoliate leave unrolled, soybean seedlings were transferred to 1/2 Hoagland solution. Stress treatments were performed as follows: for salt treatment, the roots of soybean plants were immersed in nutrient solution containing 200 mM NaCl at room temperature; for heat treatment, hydroponic seedlings were transferred into a 42°C growth chamber; for ABA treatment, soybean seedlings were grown in nutrient solution added 100 μM ABA. Root tissues were harvested after 0, 0.5, 1, 6, 12, and 24 h exposure to salt, heat, and ABA treatment. Three independent sets of control and stress treatment samples were collected, and each replicate represented a pooled sample of three individual plants. After collection, all the samples were immediately frozen in liquid nitrogen and stored at −80°C until use.

### RNA extraction and qRT-PCR analysis

The total RNA samples were isolated using Ultrapure RNA Kit (CWBIO, China). cDNA was synthesized using 2 μg RNA using the HiScript II Q Select RT SuperMix for qPCR Kit (Vazyme, China). Gene specific primers for quantitative real-time RT-PCR (qRT-PCR) analysis were designed using Primer 5.0 according to soybean cDNA sequences (Table S1). The soybean *actin 11* was used as internal reference gene. qRT-PCR reaction was performed using ChamQ SYBR qPCR Master Mix (Vazyme, China) and was conducted on ABI 7300 Real-time Detection System (Applied Biosystems, USA). The PCR reaction was carried out with the following reaction conditions: 95°C for 20 s; followed by 40 cycles of 95°C for 15 s, 60°C for 20 s and 72°C for 20 s. Samples for qRT-PCR were run in 3 biological replicates with 3 technical replicates and the data were represented as the mean ± SD (*n* = 3) for Student's *t*-test analysis. The relative gene expression was calculated using the ΔΔCt algorithm (Livak and Schmittgen, 2001; Bustin et al., 2009).

### Prokaryotic expression and purification of soybean SUMO proteins

*GmSMO1, GmSMO2*, and *GmSUMO4* genes were amplified by PCR using soybean cDNA as template. After sequencing, the amplified products were added *Sac* I and *Hind* III restriction sites to clone into the pET32a expression vector. The resultant vectors were transformed into *E. coli* BL21 cells, which were induced for expression by adding 1 mM IPTG after achieving OD600 0.6 in LB broth. Cells were collected after 4 h of growth at 28°C, and centrifuged at 4000 × g for 30 min then resuspended in PBS buffer. After sonication, the soluble protein was used directly for SDS-PAGE or purification using Ni Sepharose 6 Fast Flow resin (GE Healthcare) according to protocols in the manual.

### Protein extraction and western blot analysis

Total protein samples from three biological replicates were isolated from soybean root tissues using previously described method (Cai et al., 2017). Protein concentration was determined by Bradfrod method using BSA as the standard. 15 μg of purified GmSUMO protein and each protein sample of soybean roots was separated on 12% SDS−PAGE and transferred to nitrocellulose membrane according to standard protocols. The membrane was blocked using phosphate buffered saline (PBS) buffer containing 5% milk for 1 h at room temperature, then probed with AtSUMO1 primary antibody (AbcamInc., China, ab5316; a rabbit polyclonal antibody that reacts with ArabidopsisSUMO1/2) which was prepared in the PBS buffer containing 1% BSA (1:4000) at 4°C for overnight. After removing unbound antibodies by washing with a PBST buffer (PBS containing 0.05% Tween 20), the blot was incubated with goat antirabbit IgG secondary antibody (horseradish peroxidase conjugated, Thermo FisherScientific, USA) in the PBST buffer at a dilution of 1:20000. Chemiluminescence was carried out using the ECL Plus kit according to manufacturer's (Merck Millipore, USA) instructions.

## Results

### SUMO pathway genes in soybean

Using homology searches and domain confirmation, a total of 40 genes encoding SUMO pathway members were found in soybean, including six family groups. The detailed characteristics of each family member were shown in Table 1.

**Table 1:** Characteristics of soybean SUMO pathway genes.

**Group**	**Gene Name**	**Locus Name**	**Chromosome**	**Gene length (bp)**	**Number of Exon**	**Basic features of Protein**			**Subcellular location**
						**Length (aa)**	**MW (Da)**	**pI**	
SUMO	*GmSUMO1*	Glyma.08G320500	8	300	3	99	11,203.5	4.71	mito: 6, nucl: 5, cyto: 1, plas: 1
	*GmSUMO2*	Glyma.18G165200	18	345	3	114	12,969.1	5.58	nucl: 8, cyto: 2, chlo: 1, mito: 1, plas: 1
	*GmSUMO3*	Glyma.08G350600	8	354	3	117	13,210.6	5.68	nucl: 10, cyto: 2, chlo: 1
	*GmSUMO4*	Glyma.08G111700	8	321	3	106	12,199.7	6.67	nucl: 7, cyto: 5, extr: 2
	*GmSUMO5*	Glyma.08G111,800	8	297	3	98	11,115.8	9.14	cyto: 5, nucl: 4, chlo: 2, mito: 2
	*GmSUMO6*	Glyma.05G154000	5	297	3	98	11,144	9.59	nucl: 6, cyto: 5, mito: 2
	*GmSUMO1*	Glyma.08G320500	8	300	3	99	11,203.5	4.71	mito: 6, nucl: 5, cyto: 1, plas: 1
	*GmSUMO2*	Glyma.18G165200	18	345	3	114	12,969.1	5.58	nucl: 8, cyto: 2, chlo: 1, mito: 1, plas: 1
E1	*GmSAE1a*	Glyma.08G011400	8	846	9	281	31,131.4	5.28	extr: 11, cyto: 2
	*GmSAE1b*	Glyma.05G204000	5	996	10	331	36,839.8	5.56	nucl: 6, cyto: 6, mito: 1
	*GmSAE2a*	Glyma.13G201500	13	2,052	11	683	70,966	4.94	chlo: 4, plas: 3, nucl: 2, E.R.: 2, cyto: 1, extr: 1
	*GmSAE2b*	Glyma.12G236000	12	1,911	11	636	70,911.1	5.03	chlo: 5, plas: 3, nucl: 2, cyto: 1, vacu: 1, E.R.: 1
E2	*GmSCEa*	Glyma.11G053300	11	480	5	159	18,000.3	8.47	nucl: 7, cyto: 3, mito: 2, plas: 2
	*GmSCEb*	Glyma.01G188900	1	480	5	159	18,000.3	8.47	nucl: 7, cyto: 3, mito: 2, plas: 2
	*GmSCEc*	Glyma.17G169700	17	483	5	160	18,067.4	8.47	nucl: 10, plas: 2, cyto: 1
	*GmSCEd*	Glyma.05G091100	5	483	5	160	18,067.4	8.47	nucl: 10, plas: 2, cyto: 1
E3	*GmSIZ1a*	Glyma.12G071300	12	2,643	16	880	95,834.9	4.89	nucl: 13
	*GmSIZ1b*	Glyma.U020100	–	2,640	16	879	95,988.2	4.83	nucl: 13
	*GmSIZ1c*	Glyma.12G170900	12	2,649	16	882	96,853.1	4.75	nucl: 12, cyto: 2
	*GmSIZ1d*	Glyma.13G328100	13	2,643	17	880	96,878.1	4.78	nucl: 12, cyto: 2
	*GmMMS21*	Glyma.13G273500	13	744	7	247	27,767.8	4.74	chlo: 10.5, chlo_mito: 7, mito: 2.5
	*GmPIAL1*	Glyma.11G020900	11	2,631	16	876	95,516.7	7.54	chlo: 10, pero: 2, nucl: 1
	*GmPIAL2*	Glyma.01G222500	1	2,631	16	876	95,665.8	7.7	nucl: 7, chlo: 4, cysk: 2
SUMO Chain binding protein	*GmCBa*	Glyma.11G103800	11	618	4	205	23,015.4	9.21	nucl: 5, mito: 4, chlo: 3, cyto: 1
	*GmCBb*	Glyma.12G028700	12	618	4	205	23,149.5	9.21	chlo: 6, nucl: 4, cyto: 2, mito: 2
	*GmCBc*	Glyma.20G004500	20	405	4	134	14,955.4	8.79	Unknown
	*GmCBd*	Glyma.09G281300	9	621	4	206	23,152.8	6.5	nucl: 11, cyto: 1, mito: 1
Ubiquitin like protease	*GmPROa*	Glyma.04G193100	4	945	7	314	36,882.6	10.2	cyto: 7, chlo: 2, E.R.: 2, golg: 2
	*GmOTSa*	Glyma.18G137700	18	1,761	14	586	67,944	6.95	nucl: 13
	*GmOTSb1*	Glyma.08G287800	8	1,131	10	376	43,485.7	7.76	nucl: 13
	GmOTSb2	Glyma.08G287700	8	576	4	191	21,854.85	4.9	extr: 7, chlo: 2, cyto: 2, vacu: 2
	*GmB2a*	Glyma.06G095600	6	2,874	16	957	10,6715.9	4.19	nucl: 8, cysk: 3, plas: 1.5, golg_plas: 1.5
	*GmB2b*	Glyma.04G093800	4	2,802	16	933	104,220.9	4.24	nucl: 8, cysk: 3, plas: 1.5, golg_plas: 1.5
	*GmB2c*	Glyma.15G058100	15	1,869	10	622	70,841.3	5.46	chlo: 8, nucl: 5
	*GmB2d*	Glyma.13G256800	13	1,932	16	643	93,129.9	4.51	nucl: 5, mito: 4, chlo: 3, cysk: 1
	*GmB2e*	Glyma.02G214100	2	2,166	9	721	83,035.8	4.6	nucl: 12, cyto: 2
	*GmESD4a*	Glyma.15G148700	15	1,410	9	470	54,530.6	8.51	nucl: 6, cyto: 5, chlo: 2
	*GmESD4b*	Glyma.09G044400	9	1,407	9	468	54,626	8.88	nucl: 8, cyto: 3, chlo: 2
	*GmESD4c*	Glyma.17G027100	17	1,503	9	500	57,721.8	9	chlo: 8, nucl: 3, pero: 2
	*GmESD4d*	Glyma.07G246900	7	1,539	9	512	59,051.9	8.2	chlo: 9, nucl: 3, mito: 1

*bp, base pair; aa, amino acids; Da, Dalton; pI, isoeletricponit Subcellular predictions; chlo, (chloroplast); cyto, (cytosol); nucl, (nucleus); E.R., (endoplasmic reticulum); mito, (mitochondria); plas, (plasma membrane); extr, (extracellular); cysk, (cytoskeleton); plas, (plasma membrane); vacu, (vacuolar membrane); cyto, (cytoplasmic); pero, (peroxisomal); golg, (golgi)*.

**Figure 1:**
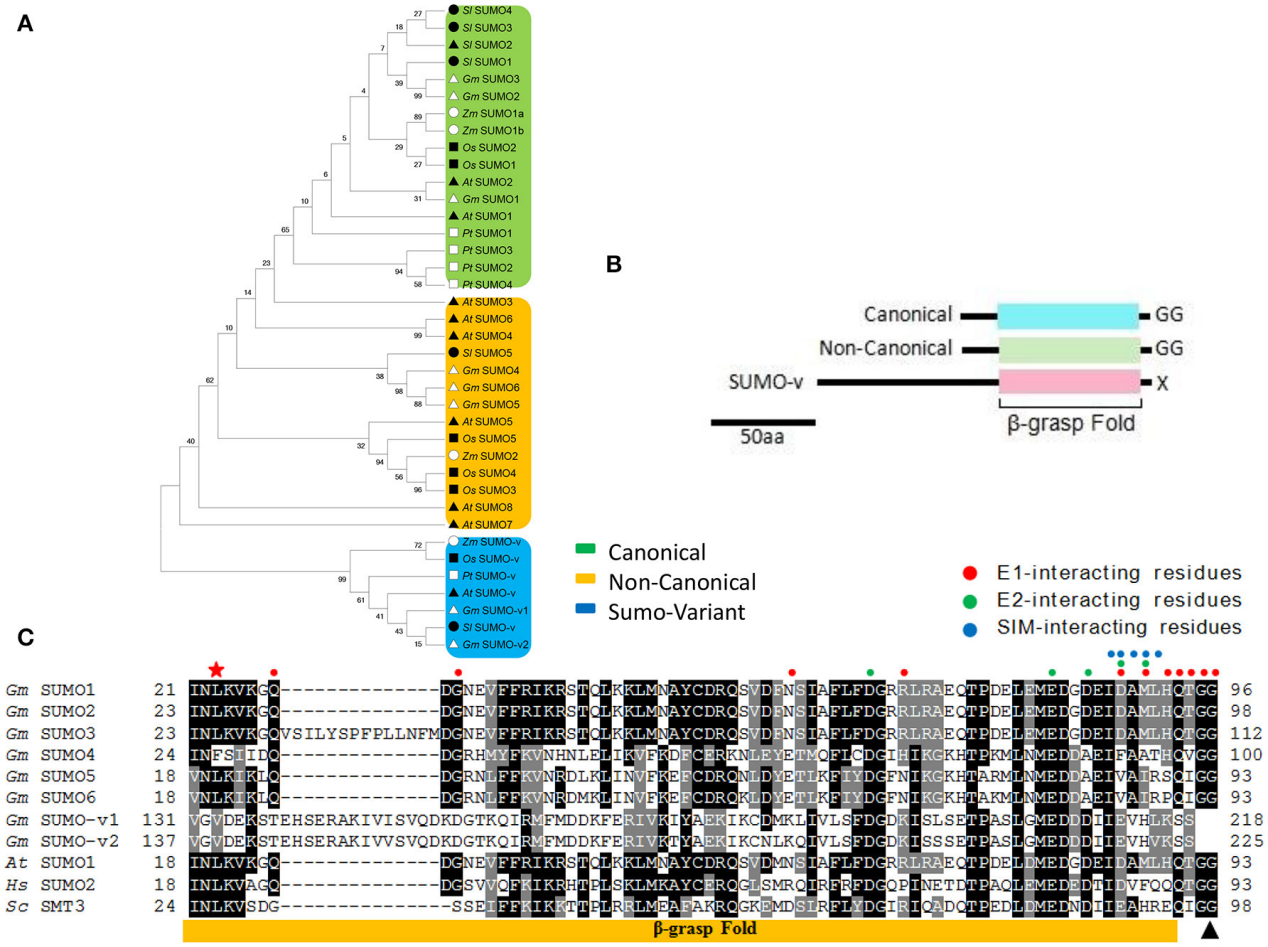
The genomes of soybean and other plant species encode a family of SUMO-related proteins **(A)** Protein sequences from *Arabidopsis thaliana, Zea mays, Oryza sativa, Solanum lycopersium, Populus trichocarpa* and *Glycine max* (Table S2) were used to construct the phylogenetic tree by the neighbor-joining method in MEGA. They were classified into three groups: canonical SUMO, non-canonical SUMO, and SUMO-variant. **(B)** Domain structures of the plant SUMO family that include the canonical and non-canonical SUMO isoforms, and SUMO-variant containing a long, conserved N-terminal extension in front of the β-grasp domain. **(C)** Alignment of SUMO sequence reveals conserved and divergent residues. Included are soybean (*Gm*) SUMO-1, -2, -3, -4, -5, -6, and SUMO-v1, -v2, along with canonical SUMOs from *Arabidopsis* (*At*SUMO1), human (*Hs*SUMO2, NP_008868.3), and yeast (*Sc*Smt3, KZV12750.1), only conserved region is shown in the figure. The yellow box locates the β-grasp-fold. The black triangle locates the processing site by ULP that exposes the diGly motif essential for conjugation in canonical SUMOs. Residues marked with red, green, and blue circle dots are important for the non-covalent binding of SUMOs to E1, E2, and SIMs (SUMO interacting motif), respectively. The asterisk denotes the conserved Lys required for forming SUMO-chains. Gray and black boxes identify similar and conserved amino acids, respectively. Dashes denote gaps.

Our soybean list included six SUMO genes, designated as *GmSUMO1* to *GmSUMO6*, all of which had three exons and encoded proteins harboring a SUMO-type β-grasp domain and predicted to localize in the nucleus (Table 1). Among these SUMO genes, *GmSUMO2* and *GmSUMO3* encoded proteins sharing 92 and 80% similarity with that from *AtSUMO1*, while *GmSUMO1* encoded a protein with 85% identity to *AtSUMO2*. By contrast, *GmSUMO4, GmSUMO5*, and *GmSUMO6* encoded products with little resemblance to either *At*SUMO1/2 or *Gm*SUMO1/2/3. To better understand the evolutionary relationship between these *SUMO* isoforms, we searched a number of other plant genomes for related sequences and constructed a phylogenetic tree (Figure 1A). Phylogenetic analysis revealed a highly conserved SUMO group including *GmSUMO1/2/3, AtSUMO1/2, ZmSUMO1a/b* and conserved representatives from other species. In contrast, a “non-canonical” group that included *GmSUMO4/5/6, AtSUMO3/5*, and *ZmSUMO2* encoded proteins shared about 30% amino acid similarities to their canonical paralogs (Figure 1A). Regardless of their dissimilarities, it is noteworthy that non-canonical members also have the C-terminal di-Gly motif necessary for conjugation (Figure 1B) which indicated their potential abilities of covalent attachment to target proteins. In support, we identified some residues important for the non-covalent binding of SUMOs to E1, E2, and SUMO-interacting motifs (SIMs, ψψXψD/S/E, D/S/EψXψψ or ψψDLT, where ψ stands for the hydrophobic amino acids and X represents any residue) conserved across the biological kingdoms (Figure 1C). Besides these identified SUMO proteins above mentioned, two longer SUMO related proteins designated as SUMO-variants (V) were detected in soybean genome (Figure 1). *Gm*SUMO-Vs contained the N-terminal extension of more than 200 residues upstream of the SUMO β-grasp domain (Table 1 and Figure 1B). Without an exposed diGly motif (Figures 1B,C), SUMO-v may not enter the well-studied E1/E2/E3 SUMOylation pathway, but probably have other functions to be explored in plants.

The initial activation of SUMOylation is adenylation of mature SUMO on the C-terminus diGly by the heterodimeric SAE1/SAE2 E1. Soybean genome expressed two small subunit SAE1 isoforms (*Gm*SAE1a/b) and two large subunit SAE2 isoforms (*Gm*SAE2a/b) (Table 1). The ubiquitin-fold domain and cysteine domain important to E1 catalysis were shown in Figure S1. After being activated by E1, the bound SUMO is transferred to an active-site cysteine in the E2 by transesterification (Miura et al., 2007a). In contrast with the single *SCE1* gene in *Arabidopsis*, the soybean genome contained four E2 genes named *GmSCEa, GmSCEb, GmSCEc*, and *GmSCEd* (Table 1). Conformed with the observations made earlier in the homologs *AtUbc9* (Kraft et al., 2005) and *OsSce1* (Nigam et al., 2008), all of these four genes were predicted to encode proteins localized predominantly to the nucleus along with SUMOs, however, whether *GmSCEs* function related to the nuclear pore complex is still not known and deserved further studies. Motif-scan analysis of *GmSCEa-d* revealed that they have highly conserved Ub-Conjugating Enzyme Catalytic (UBC) domain (spanning from 8 to 150aa residues; Figure S2A), which was common to UBC family members (Nigam et al., 2008). Phylogenetic analysis reflected that soybean *SCE* genes had high similarities with homologs in other species (Figure S2B) highlighted the conservation of E2 proteins. Collectively, we speculated that soybean homologs of SUMOylation system will be biochemically functional.

**Figure 2:**
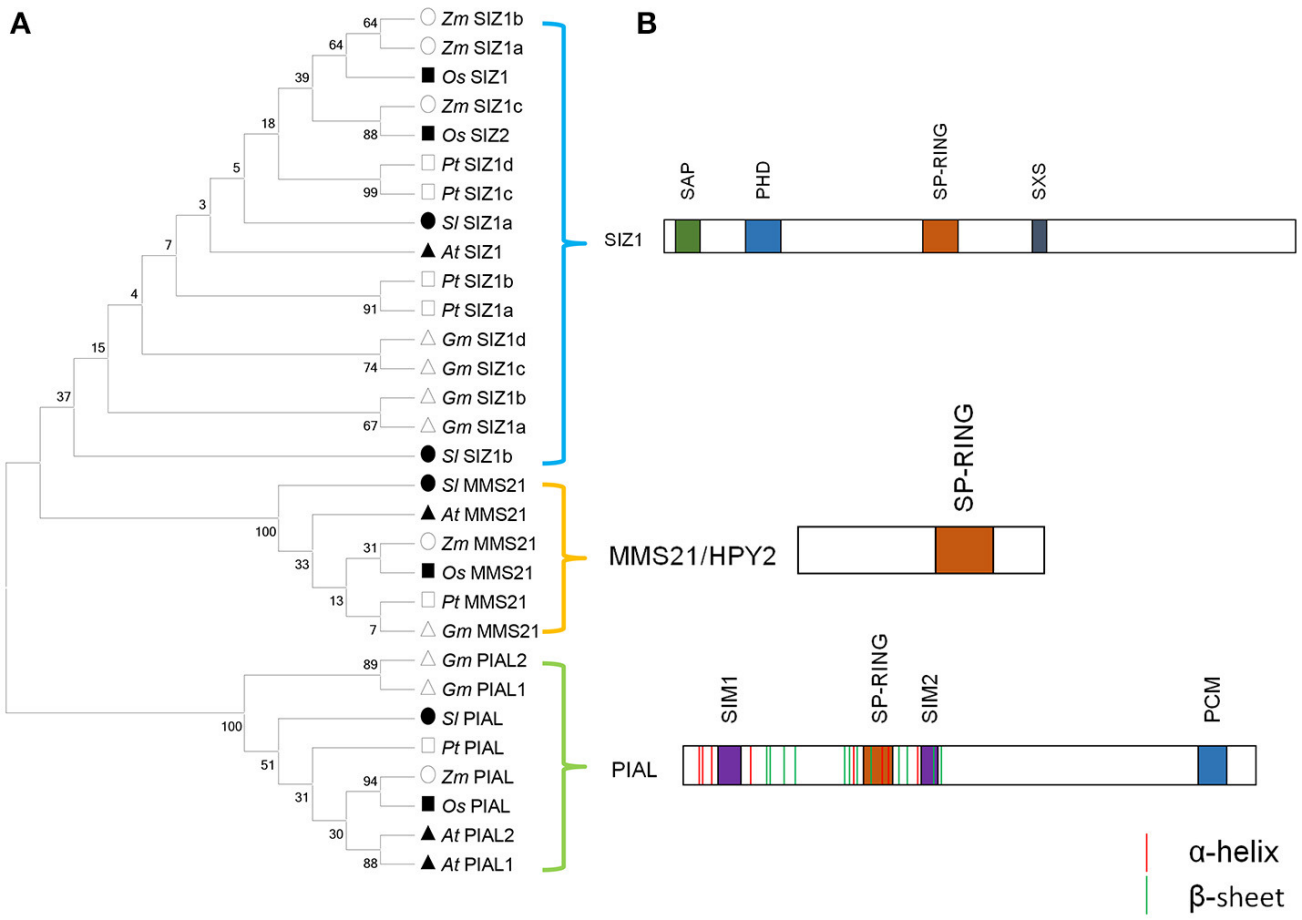
Soybean genome encodes three types of E3 ligase protein. **(A)** E3 protein sequences from *Arabidopsis thaliana, Zea mays, Oryza sativa, Solanum lycopersium, Populus trichocarpa*, and *Glycine max* (Table S2) were used to construct the phylogenetic tree. These E3 ligases can be classified into three groups. **(B)** Schematic representation of functional domains of SIZ1, HPY/MMS21 and PIAS-like type SUMO ligase.

Although SCE itself can directly transfer SUMO to substrates, E3 ligases are often required to increase the rate of SUMOylation and increase the substrate specificity during SUMOylation process (Gareau and Lima, 2010). When searching the soybean genome using E3 genes in *Arabidopsis*, we got seven soybean E3 genes (Table 1), all of which contain the conserved Siz1/PIAS(SP)-RING domain which plays the role of binding to the SUMO-E2 intermediate. These E3 genes could be divided into three groups, including SIZ1, Methyl Methane sulfonate-Sensitivity protein-21/High Ploidy-2 (MMS21/HPY2), and Protein Inhibitor of Activated STAT (PIAS)-Like (PIAL) (Figure 2A). The soybean SIZ1 group contained four proteins (*Gm*SIZ1a-d) that included the signature MIZ/SP-RING domain, as well as PINIT and SXS motifs that promoted selective SUMOylation (Figure 2B; Yunus and Lima, 2009), which was reported to be important for *Arabidopsis* SIZ1 activity (Cheong et al., 2009). Phylogenetic analysis of SIZ1 type SUMO E3 ligases from various plant species showed that *Gm*SIZ1a-d were evolutionarily most closely to *Pt*SIZ1a/b (Figure 2A).

**Figure 3:**
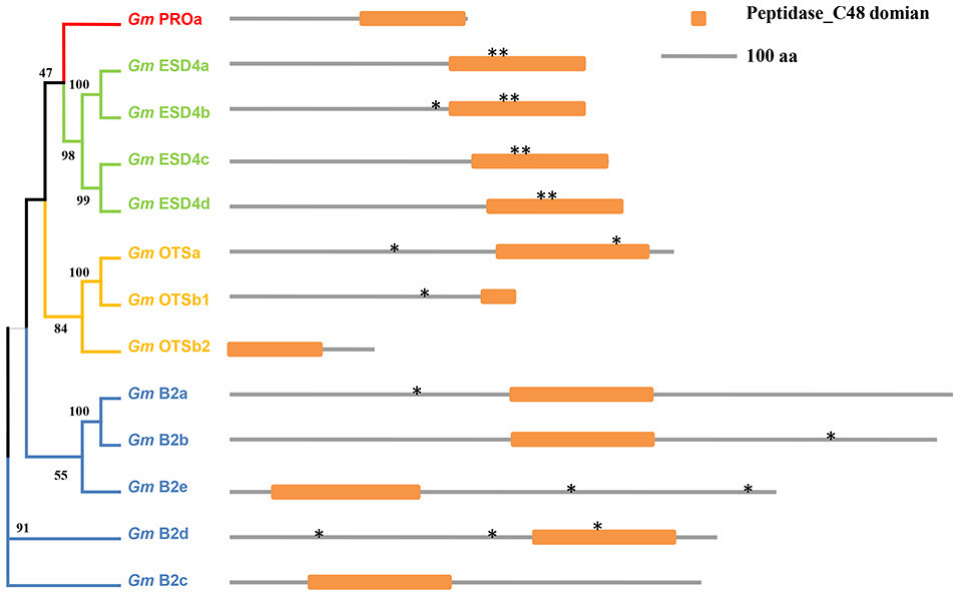
Schematic representation of functional domains of ULP family members in soybean genome. The phylogenetic analysis was carried out using MEGA (V7.0.20). Orange rectangles indicate the peptidase_C48 domain. Asterisks indicate the putative SIM motifs.

Thirteen SUMO protease genes in soybean were classified into four groups (Table 1 and Figure 3), all of which have the signature peptidase_C48 domain characterized by a catalytic triad of His-Asp-Cys that leads to peptide bond cleavage (Colby et al., 2006; Mukhopadhyay and Dasso, 2007). For class A, there was only one member in soybean, confirmed the notion that most plants encoded a single gene of this class (Figure S3; Novatchkova et al., 2012). For *Gm*ESD4a-d, several SIM sequences were identified that could bind SUMO (Figure S4). For the OTS family in soybean, the predicted *GmOTSa* encoded protein contained intact peptidase_C48 domain, whereas two loci Glyma.08G287800 and Glyma.08G287700 in soybean genome encoded N-terminal and C-terminal of the peptidase_C48 domain respectively (Figure 3), and whether either of them could exhibit SUMO protease activity is not known and deserved further study.

In addition, soybean genome contained another family of SUMO chain binding protein genes characterized by a tandem arrangement of four SIMs (Table 1 and Figure S5), which therefore have specific affinity for binding to SUMO chains. Besides, these proteins share a RING domain which was speculated to direct proteins with SUMO chains into the ubiquitin-proteasome degradation pathway (Plechanovová et al., 2011; Praefcke et al., 2012).

### *cis*-acting regulatory elements in the promoters of SUMO pathway genes

**Figure 4:**
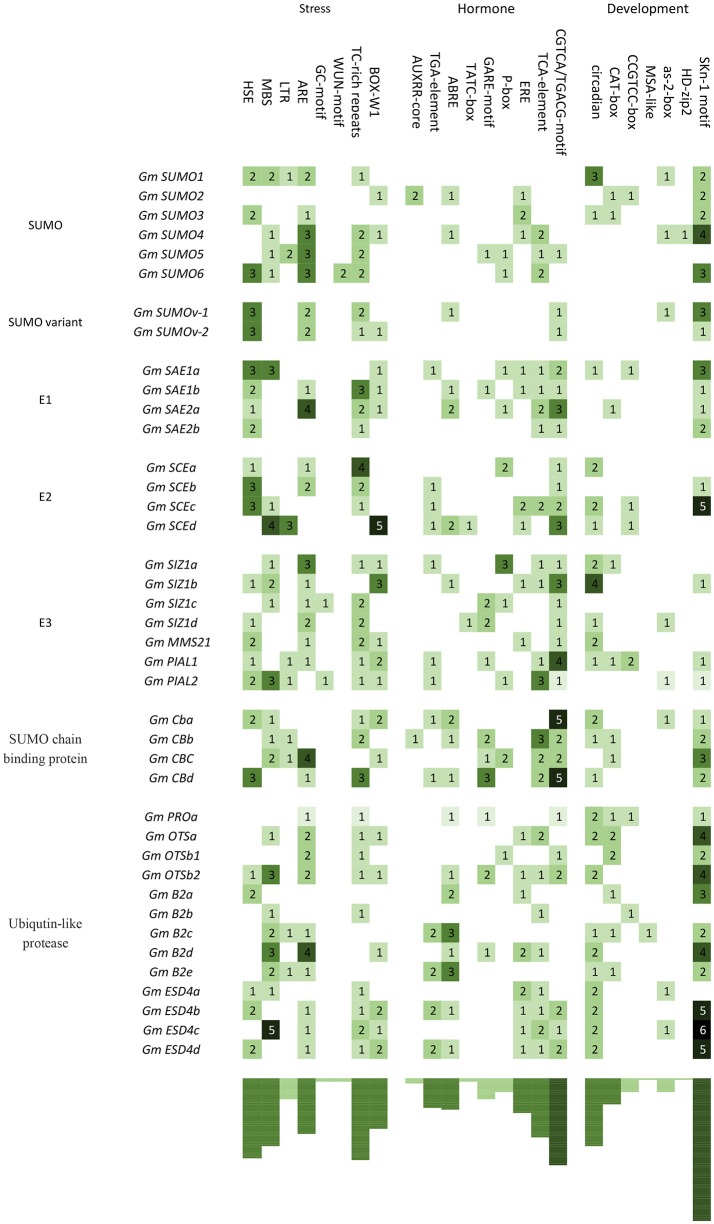
*cis*-acting regulatory elements identified in soybean SUMO pathway genes. One Thousand and five hundred blood pressure upstream sequences of 40 SUMO pathway genes were analyzed. The number and abundance of each *cis*-element are shown in the figure. Promoter sequences, *cis*-element sequences and annotation of each cis-element are available in the Tables S3,S4.

In order to get some information of stimulus induced as well as temporal and spatial specific expression patterns of these soybean SUMO pathway genes, 1,500 bp promoter regions upstream of all of these 40 soybean genes were extracted and used for *cis*-acting elements searches (Table S3). As shown in Figure 4, most genes involved in SUMOylation in soybean contained several environmental stress-related elements, including *cis*-elements that were related to plant responses to heat (HSE), drought (MBS), low temperature (LTR), anaerobic stress (ARE), and fungus (BOX-W1). Besides, several promoters contained *cis*-elements related to MeJA (CGTCA/TGACG-motif), salicylic acid (TCA-element), ABA (ABRE), GA (TATC-box, GARE-motif and P-box), auxin (AUXRR-core and TGA-element), and ethylene (ERE) (Figure 4). These results suggested that these genes were likely to be transcriptionally regulated upon environmental stresses and hormone signals. Moreover, seven types of *cis*-acting elements were involved in plant meristem (CAT-box and CCGTCC-box), endosperm (SKn-1 motif), leaf and shoot development (HD-zip2 and as-2-box), cell cycle (MSA-like) and circadian control (circadian), which covered most plant developmental stages (Figure 4). It suggested that SUMO pathway genes may be transcriptionally regulated during growth and development of soybean plants.

### Expression of SUMO pathway genes in different tissues of soybean

**Figure 5:**
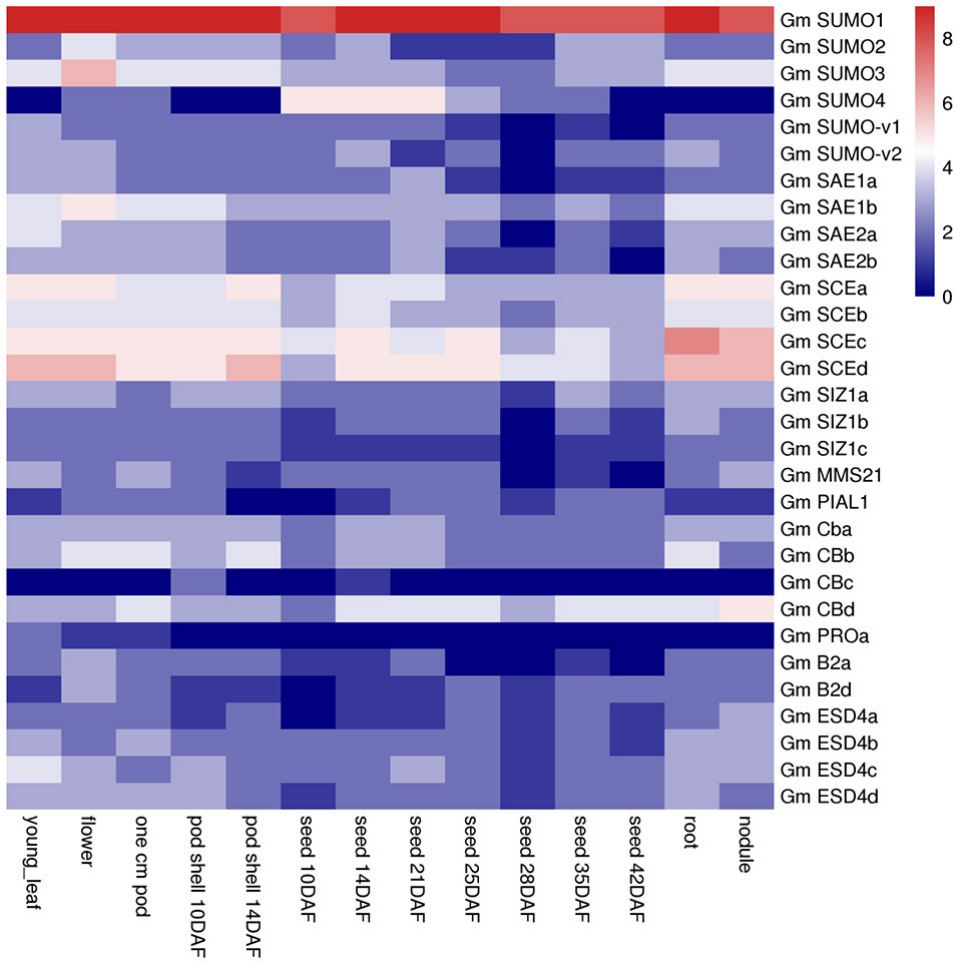
Expression pattern of soybean SUMO pathway genes in different tissues.The Reads/Kb/Million (RPKM) normalized values of expressed gene was log_2_-transformed. The abbreviation “DAF” in the tissue label indicates “Days after flowering.”

Gene functions are always closely related to where and how they are transcriptionally expressed. Transcript profiles from 14 tissues/organs were collected from soybean RNA-Seq data and downloaded from the Soybase (Table S5). A total of 30 SUMO pathway genes were expressed at a variety of developmental stages in soybean (Figure 5). Among these *SUMO* genes, *GmSUMO1* was expressed at very high levels in almost all tissues, *GmSUMO2* and *GmSUMO3* showed high expression levels in aerial and underground tissues, but much lower expression levels in seed developing stages. It suggested that *GmSUMO1* could be functional complement to the other two orthologs. In the E1 group, *GmSAE1a/b* and *GmSAE2a/b* genes exhibited relatively higher expression levels in aerial tissues, while low expression levels during seed development. Notably, all of the E2 genes showed high expression levels in all of these 14 tissues, suggesting this family of genes may support soybean normal growth and development. Interestingly, expression levels of E3 family members in some tissues were quite different. For example, expressions of *GmSIZ1c* and *GmPIAL1* were hardly detected in nearly all tissues, while *GmSIZ1a* showed relatively high expression levels in most tissues, besides, *GmSIZ1b* showed higher expression levels in flower and root, but *GmMMS21* expressed at relatively high levels in young leaf, one cm pod and nodule (Figure 5). Furthermore, our results showed that ULP family gene *GmPROa* expressed at low levels in almost all developmental stages, except the young leaf. Another interesting scenario was that *GmB2d* and *GmESD4a/b/c* were highly expressed in nodule, suggesting their potential roles in nodulation although experimental evidence is lacking for their involvement in nodule organogenesis or development. As shown in Figure 5, *GmCBa* and *GmCBb* exhibited similar expression patterns, suggesting potential redundant functions between the orthologs. Additionally, *GmCBc* was expressed at very low levels in almost all tissues, on the contrary, *GmCBd* gene showed strong expression in all of these investigated tissues. Given the results that most genes studied exhibited relatively higher expression levels in roots, the following transcriptional and translational expression profiles were performed using soybean root tissue.

### Transcriptional profiles of SUMO pathway genes in soybean roots under abiotic stress

In order to help appreciate the potential roles and transcriptional regulations of the soybean SUMO system members under abiotic stress, we examined the expression changes of 13 SUMO pathway genes in soybean roots in response to NaCl (200 mM), heat (42°C) and ABA (100 μM) treatments.

**Figure 6:**
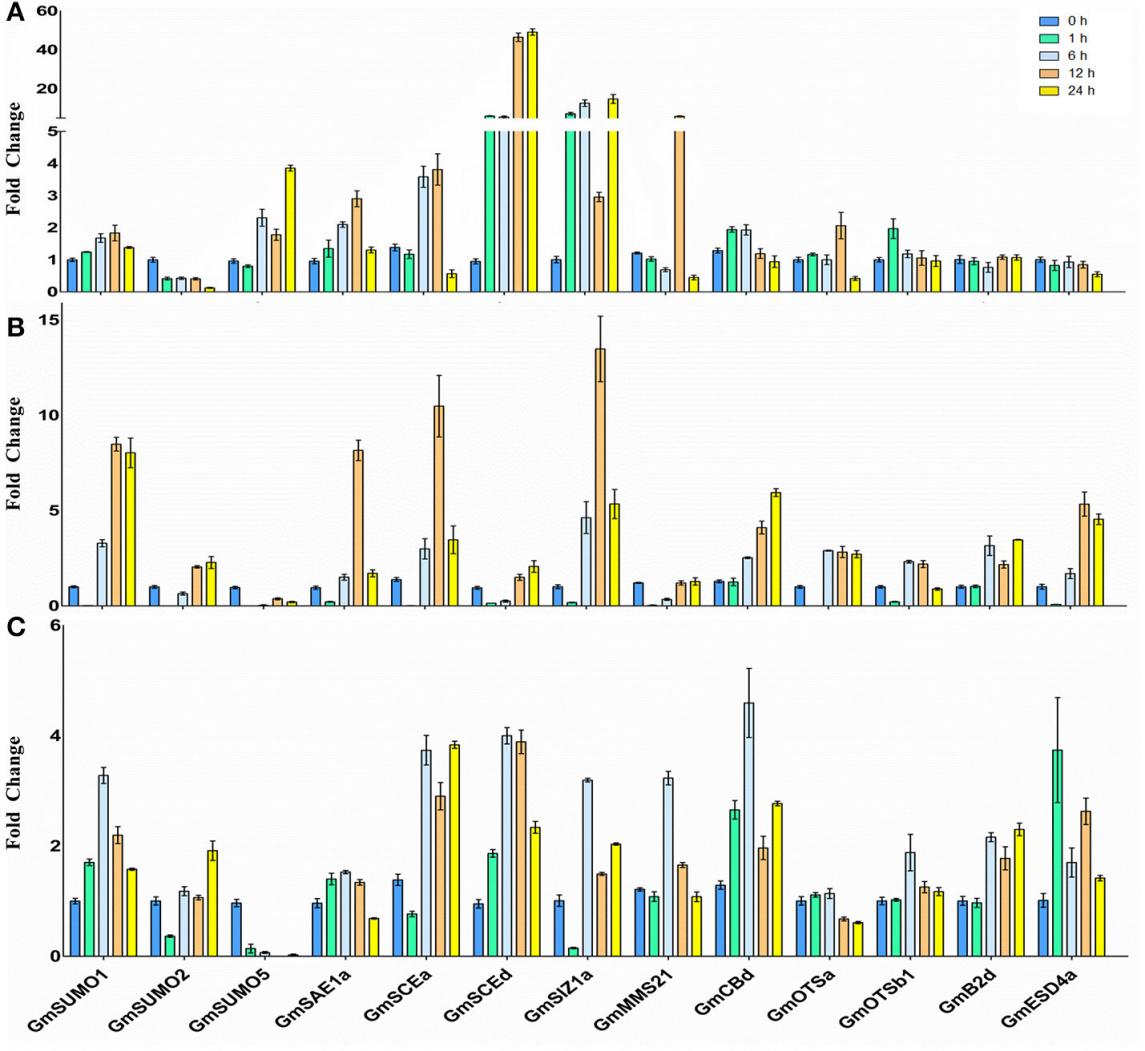
Stress induces the SUMO pathway genes expression. Changes in mRNA levels of soybean SUMO pathway genes with stress treatment were determined by quantitative real-time PCR. **(A)** NaCl (200 mM) **(B)** Heat stress (42°C) **(C)** ABA (100 μM) Changes in transcript abundance were represented as fold change by calibrating the relative mRNA levels of each time point with the relative mRNA levels of the 0 h time point.

Quantitative-PCR analysis revealed that the majority of the SUMO cascade component transcript levels were differentially regulated under salinity conditions (Figure 6A). *GmSUMO2* transcripts decreased to very low levels after exposure to salt stress, whereas, *GmSUMO5* transcripts showed up-regulated after treated for 6 h. The expression of *GmSAE1a* was slightly increased after 6 h of treatment, and was maintained nearly at initial levels after 24 h of salinity stress (Figure 6A). Among the E2 enzymes, *GmSCE1a* showed a transient increase, whereas, *GmSCE1d* transcript was significantly increased since treated for 1 h, and reached a maximum at 12–24 h which was induced about 50-fold. Among the E3 genes, *GmSIZ1a* began to increase after 1 h of treatment, whereas, *GmMMS21* showed a transient increase and decreased when treated for 24 h, which exhibited similar expression patterns with *GmOTSa* gene. These increases in gene expression may contribute to the accumulation of SUMOylated proteins after exposure to salt stress. Besides, *GmSUMO3, GmOTSb1, GmESD4a, GmB2d*, and *GmCBb* showed no detectable changes in the 24 h period after salt treatment.

When treated with heat stress, *GmSUMO1* and *GmSUMO2* were found to be induced, whereas, *GmSUMO5* showed a significant reduction under heat treatment (Figure 6B). The expression patterns of *GmSAE1a* under heat stress were similar with that under salinity treatment. Both of the E2 genes were up-regulated under heat stress, and *GmSCE1a* gene exhibited a faster response than *GmSCE1d* gene. Among these E3 genes, *GmSIZ1a* showed dramatic up-regulation 6–24 h after heat treatment; however, *GmMMS21* was significantly down-regulated 1 h after treatment, and returned to the initial levels after treated for 12 h. In addition, similar expression patterns were observed among *GmOTSa, GmESD4a*, and *GmB2d*, which showed increased expression levels from 6 h to 24 h of treatment; while *GmOTSb1* showed slightly increased from 6 h under heat treatment and after 12 h showed down-regulation. In contrast, *GmCBb* transcript showed increased gradually over the 24 h period of treatment. Notably, the majority of genes exhibited a transient decrease during the first 1 h of heat treatment, which indicated that SUMOylation may be depressed temporarily at this time point in soybean roots.

After the ABA treatment, expression of most SUMO pathway members showed slightly up-regulation with the exception of *GmSUMO2, GmSAE1a, GmOTSa*, and *GmOTSb1* genes which were maintained nearly basal levels during the 24 h period of treatment (Figure 6C). *GmSUMO1* gene showed a transient positive response 6 h after ABA treatment and subsequently returned to initial levels. Conversely, *GmSUMO5* transcript decreased significantly, thereby indicating a specific role for *GmSUMO1* in ABA stress response. All of the E2 and E3 family genes transcript levels increased after 6 h of treatment, and *GmMMS21* showed a transient up-regulation followed by restoration to basal levels. Among the ULP family, *GmESD4a* transcript showed up-regulation 1 h after the ABA treatment, indicating that *GmESD4a* may be responsible for a quick de-SUMOylation process after ABA treatment.

### SUMOylation profiles in soybean roots under abiotic stress

In order to explore the regulation of SUMOylation in response to abiotic stress in soybean roots, western blot analysis was performed to monitor the expression changes on the translational level. To test whether anti-AtSUMO1 antibody could detect soybean SUMOs, *GmSUMO1, GmSUMO2*, and *GmSUMO4* genes were amplified (Table S6) and cloned into pET32a expression vector for protein expression. The western blot analysis indicated that anti-AtSUMO1 antibody was able to specifically detect GmSUMO1 and GmSUMO2 proteins, but not for GmSUMO4 (Figure S6).

**Figure 7:**
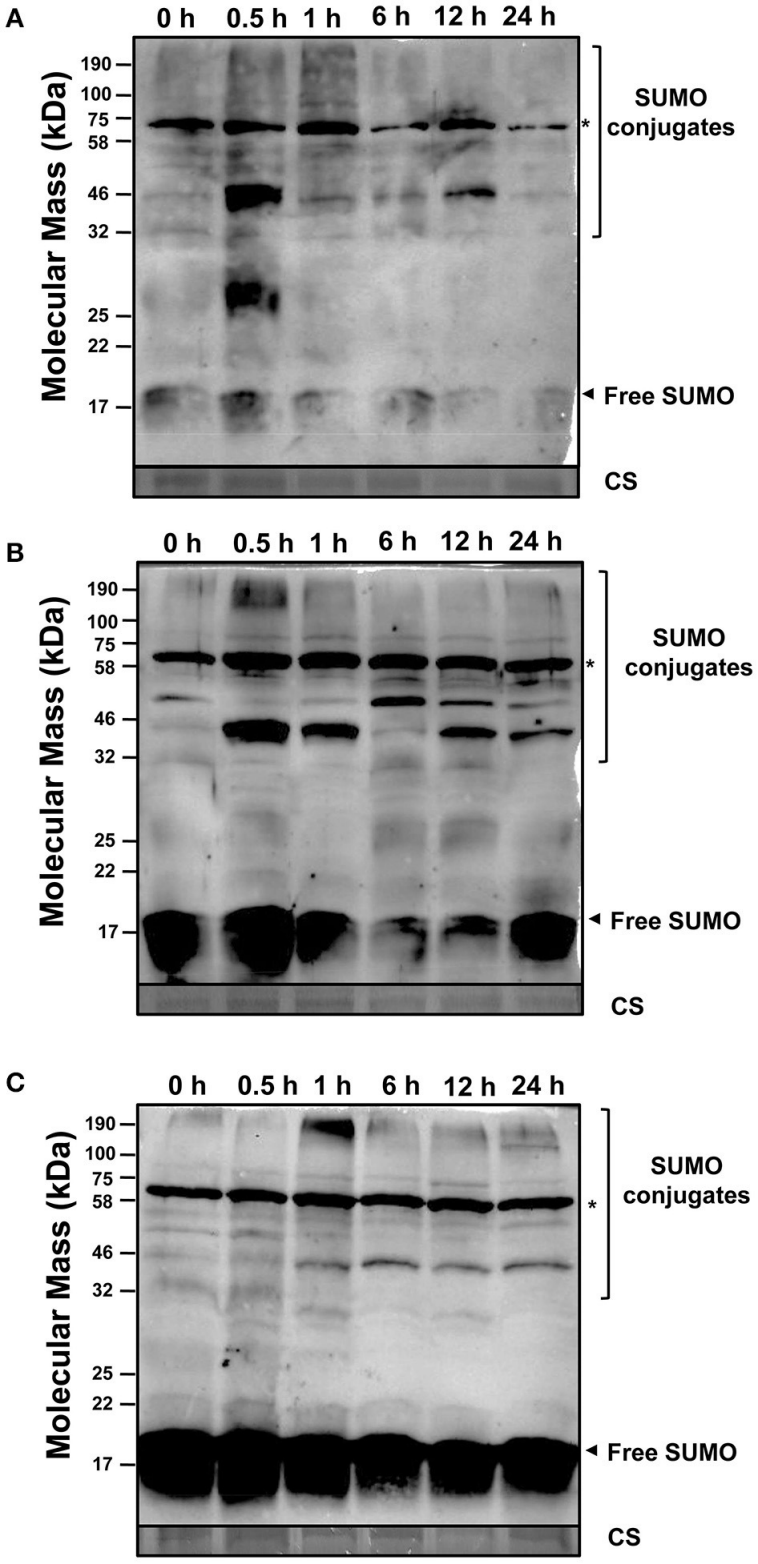
SUMOylation in soybean root is induced by stress. Fifteen microgram of total proteins extracts from the soybean roots at the second trifoliolate leaf stage were subjected to western blot analysis against anti-AtSUMO1. Coomassie stained of high abundant protein showed equal loading of protein samples. **(A)** NaCl (200 mM) **(B)** Heat stress (42°C) **(C)** ABA (100 μM). Free SUMO and conjugates are highlighted by the arrowheads and brackets respectively. The asterisk denotes a non-inducible immunoreactive product.

During salt treatment, roots accumulated large amount of low molecular weight SUMO conjugates as soon as 0.5 h after salt stimulus (Figure 7A), and the accumulation of high molecular weight SUMOylated products was detected from 0.5 to 1 h of salt treatment. At the same time, the level of free SUMO was greatly reduced from 1 h of treatment, and when treated for 24 h, most free SUMOs have been exhausted in soybean roots. Consistent with the results got from poplar (Reed et al., 2010), SUMOylation by salinity treatment in soybean roots was rapidly inducible and probably has some functional implications.

Rapid stimulation of SUMOylation in response to heat shock had been reported in several plant species (Chaikam and Karlson, 2010; Lopez-Torrejon et al., 2013; Augustine et al., 2016). In this study, SUMO conjugates were detected to be accumulated as soon as 0.5 h after heat stress, which was conformed with previous studies (Cai et al., 2017). Accordingly, the free SUMO pool showed decreased after 1 h of heat shock, and restored to nearly the original level after 24 h of treatment (Figure 7B).

After treating with ABA, the high molecular weight SUMO conjugates accumulated significantly at 1 h after treatment, but slightly decreased at later time points, and increased from 12 to 24 h after treatment (Figure 7C), which indicated that ABA treatment effected SUMOylated protein accumulation in soybean roots. These above results further supported the hypothesis that the rapid protein SUMOylation was an essential functional component in response to abiotic stress in soybean.

## Discussion

Previous studies in *Arabidopsis* connected the protein SUMOylation to plant development and defense against environmental challenges (Castro et al., 2012). Thus, manipulation of this post-translational modification system might provide opportunities to modulate the plant stress resistance with lower inputs. However, only a few examinations have been made of SUMO pathway members in soybean (Cai et al., 2017). In this study, 40 SUMO pathway genes in soybean were identified. The soybean genome contained gene models for SUMO and its respective conjugation and de-conjugation machinery including E1, E2, E3, and SUMO protease components that were closely related to homologs in *Arabidopsis*. Transcriptional and translational expression profiling of soybean SUMOylation system allowed us to identify the cascade members that have crucial roles in the soybean responses to salt, heat and ABA stresses, and explain the role of SUMOylation in soybean abiotic stress responses.

Compared with the eight putative SUMO copies in *Arabidopsis* genome, soybean genome contained six members shared a similar ubiquitin-like structural conformation characterized by a β-grasp fold. In soybean, three canonical SUMO genes were present that expressed polypeptides homologous to canonical members in other eukaryote species, which have been well recorded to conjugate to a variety of target proteins in response to environmental stimuli (Kurepa et al., 2003; Saracco et al., 2007). Among these SUMO genes in soybean, *GmSUMO1* was constitutively expressed in most organs and seed developmental stages according to the RNA seq data (Figure 5), which showed similar expression patterns with the counterpart genes in *Arabidopsis* (van den Burg et al., 2010). On the contrary, the expression of non-canonical SUMO *GmSUMO4* in soybean appeared relatively weak based on transcriptional analysis. Moreover, *GmSUMO1* transcript showed a sustained up-regulation 1 h after ABA treatment, while *GmSUMO2* and -*5* were not induced (Figure 6), suggesting a specific role of *GmSUMO1* in ABA response. Based on the high sequence similarity and overlapping expression profiles, we speculated that *GmSUMO1, -2*, and *-3* might be functional redundant, while whether the biological functions of *GmSUMO1*/*2*/*3* were essential as their homologs in *Arabidopsis* still need to be identified. The roles of SUMOs under abiotic stress were inferred by the detection of increasing SUMOylation during stress (Catala et al., 2007; Conti et al., 2008; Golebiowski et al., 2009; Miura et al., 2011). In this study, rapid regulations of SUMO-conjugates in soybean roots were identified upon salinity, heat and ABA elicitation (Figure 7). Although most target proteins bear a single SUMO moiety, some are modified with a poly-SUMO chain onto a single Lys in the substrate. Previous study demonstrated that the residue Lys10 in *Arabidopsis* SUMO2 was required for self-SUMOylation to form the multimeric chains *in vitro* (Colby et al., 2006). It can be deduced that soybean SUMO proteins except for GmSUMO4 may be able to form poly-SUMO chains, given the existence of conserved Lys residue in these soybean SUMO proteins (Figure 1C). Notably, there were indeed SUMOylated products with the molecular mass of approximately 30 and 45 kDa in soybean SUMOylation profiles (Figure 7), which were speculated to be potential SUMO dimers and trimers in soybean. The poly-SUMO chain was reported to serve as degradation signal, channeling chain-modified target proteins into degradation by SUMO chain binding ubiquitin ligases (Plechanovová et al., 2011; Praefcke et al., 2012). Thus, the appearance of SUMO dimers and trimers in soybean was supposed to be an early response to stimuli that promoted reallocation of SUMO moieties to target proteins.

Besides SUMOs, soybean genome encoded all components participate in SUMOylation and de-SUMOylation cascade. The SUMO-activating enzyme E1 (SAE) enzymes facilitate conjugation of SUMO proteins through a series of reactions including adenylation, formation of E1-SUMO thioester and transfer of thioester from E1 to E2 conjugating proteins (Lois and Lima, 2005). Previous study has demonstrated that SAE2 formed a tight heterodimer with SAE1 and controlled the localization of the SUMO SAE heterodimer to the nucleus (Truong et al., 2012). In this study, nuclear locations of SAE subunits were also found in soybean genome (Table 1). In *Arabidopsis*, loss-of-function mutant *sae2-1* exhibited developmental arrest at the early stages of embryogenesis, suggesting the essential role of SAE2 (Saracco et al., 2007). Given the comparable expression patterns between SAE1a and SAE1b, SAE2a and SAE2b (Figure 5), we suggested that there may be functionally redundancy between these paralogous SAE polypeptide. Strikingly, *GmSAE1a* gene was found to be enhanced by eight-fold after heat treatment for 12 h, which was presumed to contribute to the accumulation of SUMO-conjugates in responding to unfavorable environment.

GmSCEs belongs to conjugating enzyme family, characterized by a conserved UBC domain that spans most of the protein. In soybean, four *sce* genes showed high similarity at the amino acid levels (Figure 2), which indicated that these orthologous genes may be a result of duplication events. Putative *cis*-elements in 1.5 kb region upstream of *GmSCE* genes possessed higher abundance of stress-related elements like HSE, MBS, ARE, TC-rich repeats and CGTCA/TGACG-motif, which provided useful cues for determining the responsiveness of *GmSCE* genes to environmental stimuli. Indeed, our analyses showed that *GmSCE* transcripts were significantly up-regulated under heat, salt and ABA treatments, which was in conformity with the observations made earlier in rice (Nigam et al., 2008). Recent study provided evidence that overexpression of *SaSce9* gene from a grass halophyte in plant can improve salinity and drought stress tolerance demonstrated the potential of using this gene in crop improvement (Ratna and Prasanta, 2012). Thus, biological functions of *GmSCEa* and *GmSCEd* under abiotic stress are deserved further study.

Besides, soybean genome encoded three types of SUMO E3 ligases containing the signature SP-RING domain (Figure 2). The functions of E3 ligases in the regulation of environmental stress responses and developmental processes have been extensively characterized in *Arabidopsis* (Lois, 2010; Miura and Ohta, 2010) and rice (Li et al., 2013; Mishra et al., 2017). Recently, two identical SIZ1 homologs in soybean, *GmSIZ1a* and *GmSIZ1b*, were identified, which were reported to mediate SUMO modification of nuclear proteins and regulate vegetative growth in soybean (Cai et al., 2017). In this study, *SIZ1* gene in soybean was significantly up-regulated in response to heat and salt stress treatments (Figure 6). However, whether *GmSIZ1a/b* could regulate environmental stress responses in soybean is still needed to be determined. The second type of E3 ligases *MMS21* was reported to function as an important regulator of cell proliferation and cytokinin in signaling during root development in *Arabidopsis* (Huang et al., 2009). Previous work has reported that *Arabidopsis* plants exposed to ABA accumulate increased levels of SUMOylated proteins dependent on MMS21 activity, because the accumulation of SUMO conjugates is significantly lower in *mms21* mutant, and higher in MMS21-OE compared with WT plants (Zhang et al., 2013). In this study, we found that the expression levels of *GmMMS21* gene were induced by ABA and salt treatment (Figure 6), it was presumed that *GmMMS21* gene could act a pivotal part in salt stress response in soybean by regulating gene expression in the ABA-dependent manner. Moreover, there is another type of E3 ligases containing SP-RING domain and SUMO interaction motifs (SIM) which designated as PIAL1 and 2 that function as SUMO ligases specialized in SUMO chain formation. Mutant studies in *Arabidopsis* indicated that *PIAL1* and *2* functioned in the regulation of salt and osmotic stress responses by removal of SUMOylation substrates, and may contribute to connection of SUMOylation pathway to ubiquitin proteolytic pathway (Tomanov et al., 2014). However, the biological functions and molecular mechanisms of PIAL1 and 2 in SUMOylation cascade are still largely unknown in soybean. The up-regulation of most of E2 and E3 genes in soybean under NaCl, heat and ABA stimuli (Figure 6) may account for stress induced accumulation of SUMO conjugates which detected by western blot analysis (Figure 7).

Compared with other SUMO pathway components, there are a larger number of plant SUMO proteases (Figure 3) which cleave precisely between the terminal Gly of SUMO and the substrate Lys, and play a vital regulatory role during de-SUMOylation (Hickey et al., 2012). So far only a few bona fide SUMO proteases like ESD4 and OTS1/OTS2 have been characterized using genetic, physiological, and biochemical approaches. The *Arabidopsis esd4* mutant showed extreme early flowering and alterations in shoot development, suggesting that SUMOylation played roles in plant development which regulated by ESD4 (Murtas et al., 2003). Previous study suggested that the OTS class of ULP-like SUMO proteases may provide routes for boosting crop growth under stress because over-expression of *OTS* gene led to salt tolerance (Conti et al., 2008). However, SUMO proteases remain largely unstudied, especially in soybean and other crop plants. Similar with *AtOTS1, OTS* gene expression was enhanced in soybean under heat and salt stress would suggest that soybean stimulated the de-SUMOylation process in response to abiotic stresses, which had been shown in the soybean SUMOylation profiles (Figure 7).

## Conclusions

In summary, a total of 40 genes involved in SUMOylation system from the soybean genome were identified. We investigated the classification, characterization and tissue-specific expression of these SUMO pathway genes, revealing SUMO pathway members broadly participate in the regulation of plant tissue development. Meanwhile, qRT-PCR analysis to 13 SUMO pathway genes suggested that these genes functioned at the transcriptional level during adaption to adverse environments. Moreover, the investigation of SUMOylation profiles in response to salt, heat and ABA treatment demonstrated that soybean responded to abiotic stress treatments by regulating the SUMO conjugated proteins. These results may contribute to the existing knowledge on the complexity of soybean post translational modifications that occur in response to environmental stresses. Further proteome wide identification of various targets will be paramount to defining how SUMOylation ultimately participates in stress protection.

## Author contributions

YL performed the analysis and laboratory assays and wrote the manuscript. GW and ZX performed the phylogentic analysis and the RNA-seq analysis. JL, MS, and JG provide help in analysis of qRT-PCR and western blot. WJ conceived and designed the experiments, facilitated the project, and assisted in manuscript preparation. All authors read and approved the final manuscript.

### Conflict of interest statement

The authors declare that the research was conducted in the absence of any commercial or financial relationships that could be construed as a potential conflict of interest.

## Supplementary material

Figure S1 Sequence alignment of SAE2s from *Arabidopsis* and soybean. Adenylation, cysteine, and ubiquitin-fold domains are displayed below the sequence. Red asterisk indicates the catalytic cysteine residue. Blue asterisk indicates the predicted SUMOylation site. Gray and black boxes identify similar and identical amino acids, respectively. Dashes denote gaps.

Figure S2 Soybean genome encodes four E2 genes. **(A)** Structure-based sequence alignment of *Gm*SCEa/b/c/d along with SCEs from *Arabidopsis* and rice reveals conserved domain and motif. UBCC (Ub-Conjugating Enzyme Catalytic) domain is indicated by dotted line. Asterisk highlights the activate-site Cys. Green rectangles and blue arrows indicate helices and strands. Gray and black boxes identify similar and conserved amino acids, respectively. Dashes denote gaps. **(B)** E2 protein sequences from *Arabidopsis thaliana, Zea mays, Oryza sativa, Solanum lycopersium, Populus trichocarpa* and *Glycine max* (Table S2) were used to construct the phylogenetic tree by the neighbor-joining method in MEGA.

Figure S3 Phylogenetic tree of full-length ULP proteins from soybean and other plants. ULP family protein sequences from *Arabidopsis thaliana, Zea mays, Oryzasativa, Solanum lycopersium, Populus trichocarpa* and *Glycine max* (Table S2) were used to construct the phylogenetic tree. These ULPs can be classified into four groups.

Figure S4 Sequence alignment of the *Gm*ESD4s. Red bars above the sequences highlight predicted SIM motif. Asterisks indicate SUMOylation sites. The extent of the Peptidase_C48 domain is indicated below the sequence. Green arrowheads indicate the His-Asp-Cys catalytic triad. Gray and black boxes identify similar and conserved amino acids, respectively. Dashes denote gaps.

Figure S5 Alignment of SUMO chain binding proteins in soybean. RING finger domain is indicated by the dotted box. Red lines indicate the SIM motifs. Gray and black boxes identify similar and conserved amino acids, respectively. Dashes denote gaps.

Figure S6 Detection of soybean SUMO proteins using anti-AtSUMO1 antibody. **(A)** SDS-PAGE analysis of His:GmSUMO1/2/4 fusion protein expressed in *E. Coli* strain BL21. M, protein marker; 1, pET32a vector control; 2, pET32a-GmSUMO1; 3, pET32a-GmSUMO2; 4, pET32a-GmSUMO4. **(B)** SDS PAGE analysis of purified His:GmSUMO1/2/4 fusion protein using Ni-NTA. M, protein marker; 1, pET32a vector control; 2, pET32a-GmSUMO1; 3, pET32a-GmSUMO2; 4, pET32a-GmSUMO4. **(C)** Western-blot analysis of purified GmSUMO1/2/4 protein against anti-AtSUMO1 antibody. M, protein marker; 1, pET32a vector control; 2, GmSUMO1 protein; 3, GmSUMO2 protein; 4, GmSUMO4 protein.

Table S1 Primers used for quantitative RT-PCR of soybean SUMO pathways genes.

Table S2 SUMO pathway genes in six species.

Table S3 Sequence of 1,500 bp upstream of SUMO pathway genes in soybean.

Table S4 Detailed information of cis-acting element.

Table S5 Normalized data (Reads/kilobase/million normalization of the raw data) of SUMO pathway genes in soybean.

Table S6 CDS sequence and amplified primers for GmSUMO1/2/4 genes.
