# Supplementary figures and images for: Organization and Regulation of Soybean SUMOylation System under Abiotic Stress Conditions
Source: Front Plant Sci. 2017 Aug 21;8:1458. doi: 10.3389/fpls.2017.01458 (PMC5573446; doi:10.3389/fpls.2017.01458)

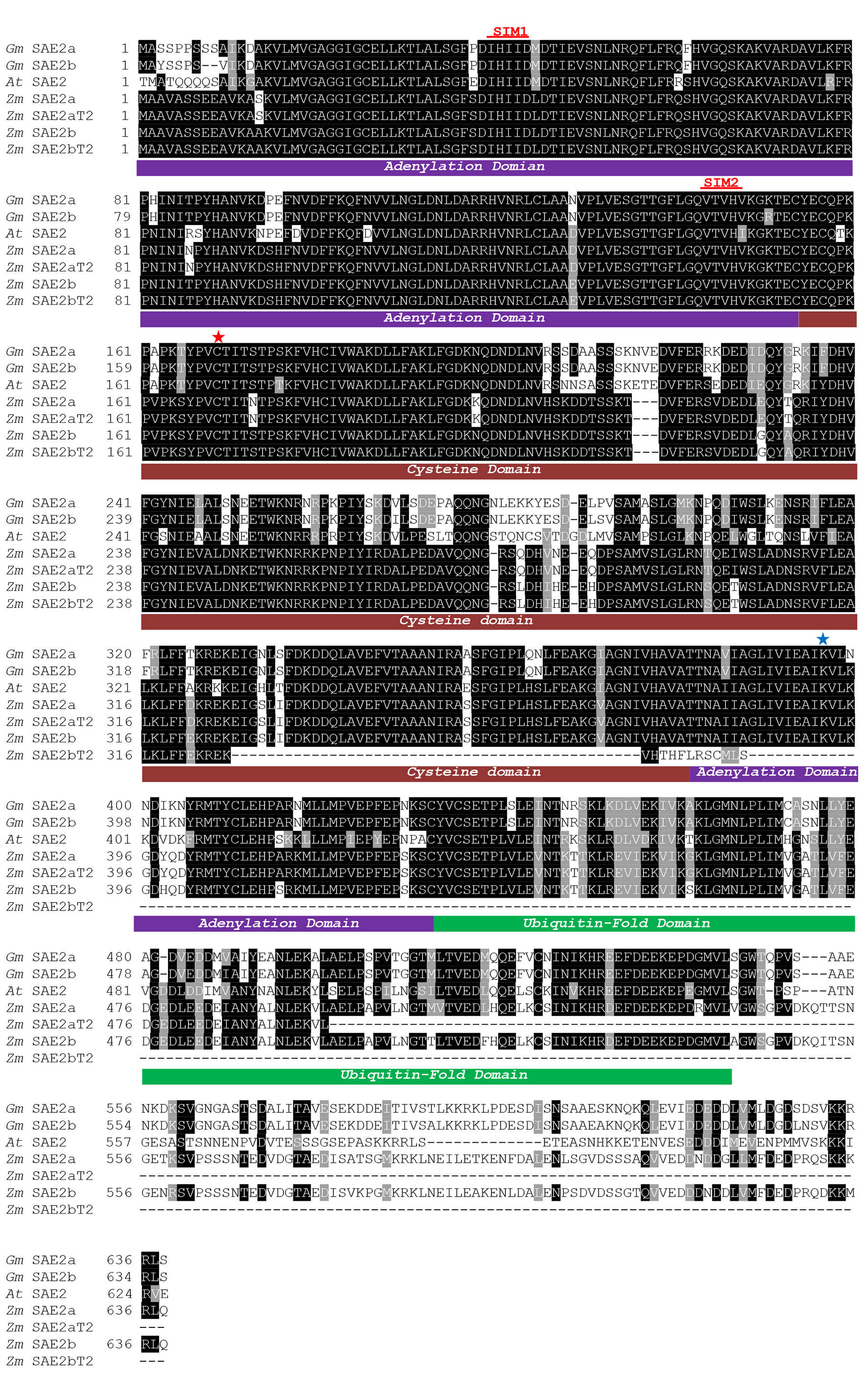

Supplement: Figure S1 — Sequence alignment of SAE2s from Arabidopsis and soybean. Adenylation, cysteine, and ubiquitin-fold domains are displayed below the sequence. Red asterisk indicates the catalytic cysteine residue. Blue asterisk indicates the predicted SUMOylation site. Gray and black boxes identify similar and identical amino acids, respectively. Dashes denote gaps. [file Image1.TIF]

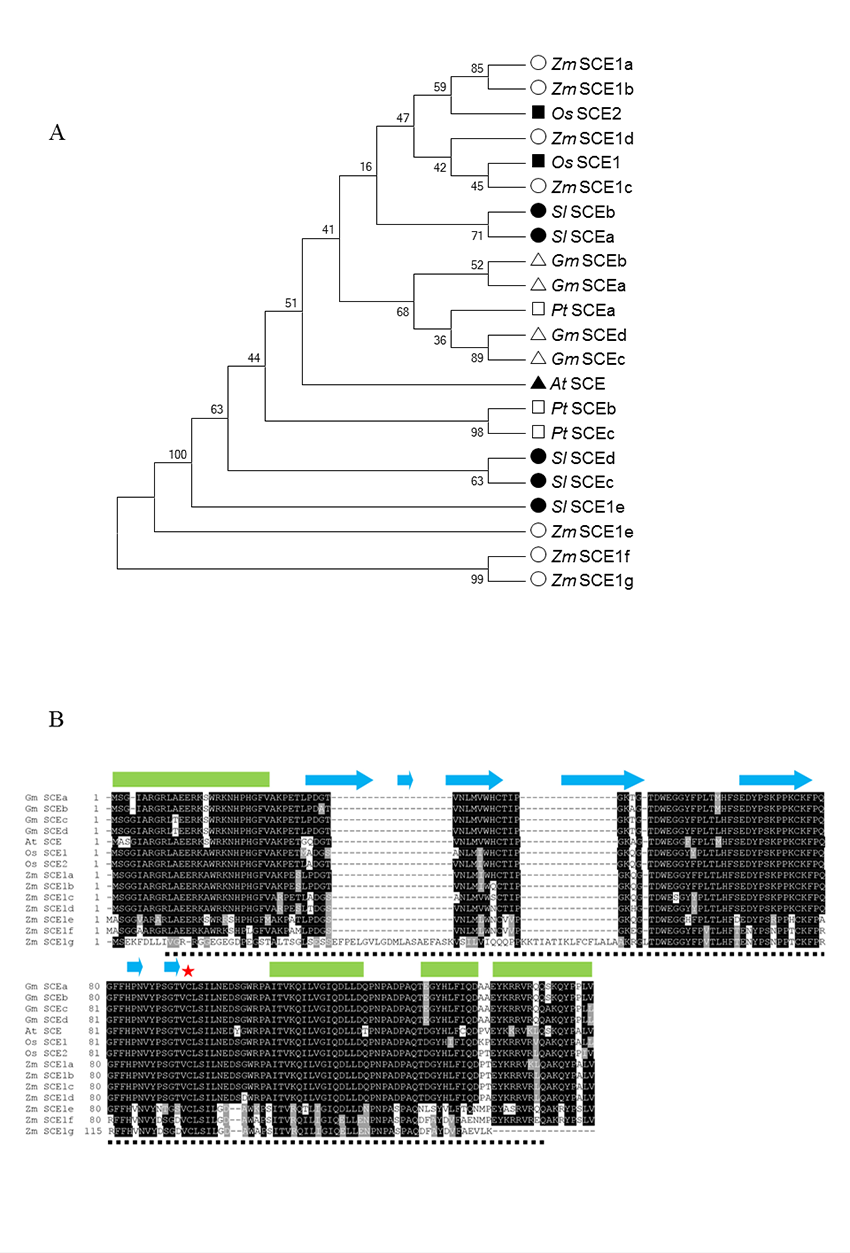

Supplement: Figure S2 — Soybean genome encodes four E2 genes. (A) Structure-based sequence alignment of GmSCEa/b/c/d along with SCEs from Arabidopsis and rice reveals conserved domain and motif. UBCC (Ub-Conjugating Enzyme Catalytic) domain is indicated by dotted line. Asterisk highlights the activate-site Cys. Green rectangles and blue arrows indicate helices and strands. Gray and black boxes identify similar and conserved amino acids, respectively. Dashes denote gaps. (B) E2 protein sequences from Arabidopsis thaliana, Zea mays, Oryza sativa, Solanum lycopersium, Populus trichocarpa and Glycine max (Table S2) were used to construct the phylogenetic tree by the neighbor-joining method in MEGA. [file Image2.TIF]

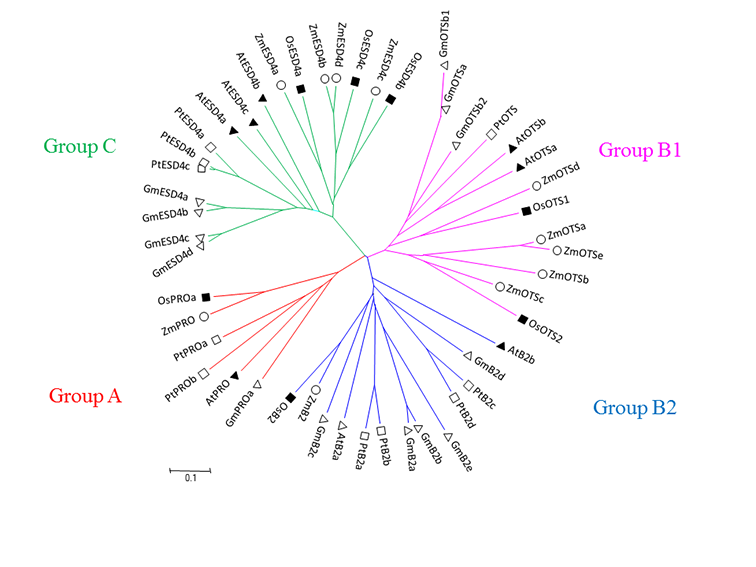

Supplement: Figure S3 — Phylogenetic tree of full-length ULP proteins from soybean and other plants. ULP family protein sequences from Arabidopsis thaliana, Zea mays, Oryzasativa, Solanum lycopersium, Populus trichocarpa and Glycine max (Table S2) were used to construct the phylogenetic tree. These ULPs can be classified into four groups. [file Image3.TIF]

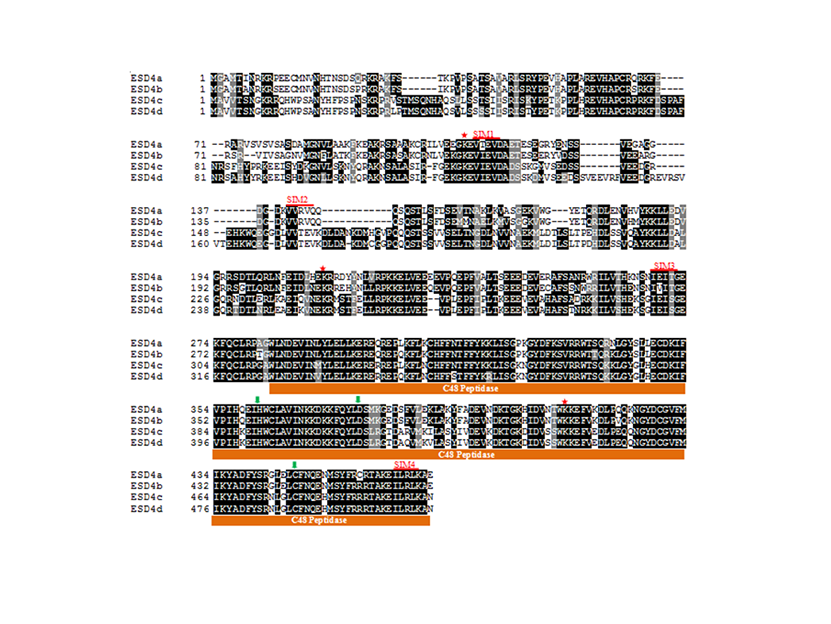

Supplement: Figure S4 — Sequence alignment of the GmESD4s. Red bars above the sequences highlight predicted SIM motif. Asterisks indicate SUMOylation sites. The extent of the Peptidase_C48 domain is indicated below the sequence. Green arrowheads indicate the His-Asp-Cys catalytic triad. Gray and black boxes identify similar and conserved amino acids, respectively. Dashes denote gaps. [file Image4.TIF]

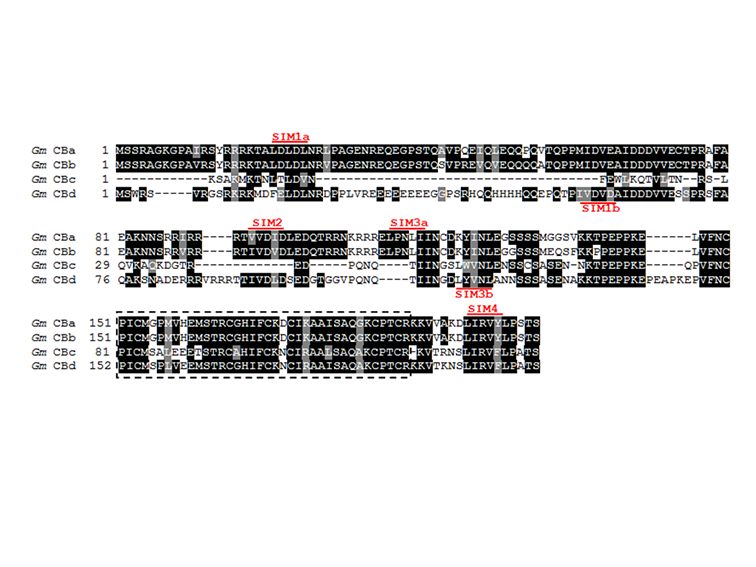

Supplement: Figure S5 — Alignment of SUMO chain binding proteins in soybean. RING finger domain is indicated by the dotted box. Red lines indicate the SIM motifs. Gray and black boxes identify similar and conserved amino acids, respectively. Dashes denote gaps. [file Image5.TIF]

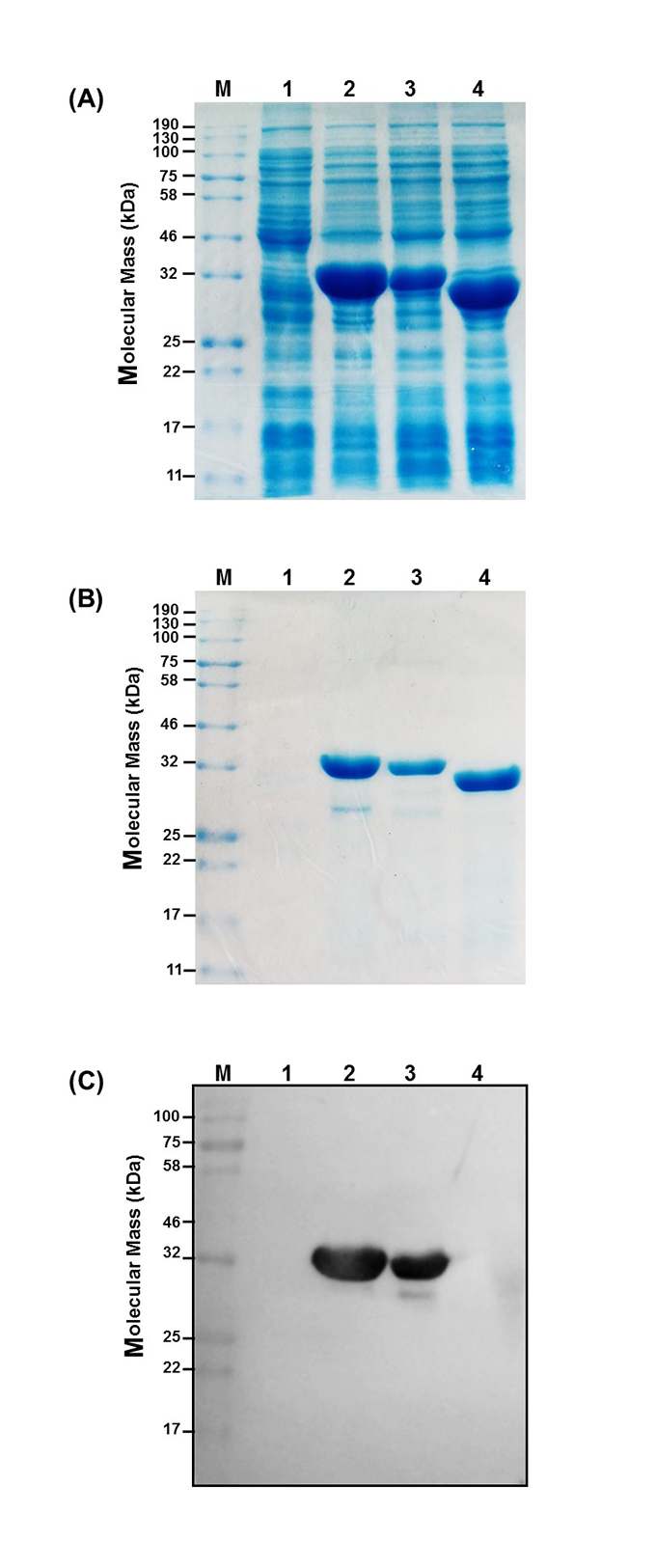

Supplement: Figure S6 — Detection of soybean SUMO proteins using anti-AtSUMO1 antibody. (A) SDS-PAGE analysis of His:GmSUMO1/2/4 fusion protein expressed in E. Coli strain BL21. M, protein marker; 1, pET32a vector control; 2, pET32a-GmSUMO1; 3, pET32a-GmSUMO2; 4, pET32a-GmSUMO4. (B) SDS PAGE analysis of purified His:GmSUMO1/2/4 fusion protein using Ni-NTA. M, protein marker; 1, pET32a vector control; 2, pET32a-GmSUMO1; 3, pET32a-GmSUMO2; 4, pET32a-GmSUMO4. (C) Western-blot analysis of purified GmSUMO1/2/4 protein against anti-AtSUMO1 antibody. M, protein marker; 1, pET32a vector control; 2, GmSUMO1 protein; 3, GmSUMO2 protein; 4, GmSUMO4 protein. [file Image6.TIF]
